# Ensemble Fuzzy Feature Selection Based on Relevancy, Redundancy, and Dependency Criteria

**DOI:** 10.3390/e22070757

**Published:** 2020-07-09

**Authors:** Omar A. M. Salem, Feng Liu, Yi-Ping Phoebe Chen, Xi Chen

**Affiliations:** 1 School of Computer Science, Wuhan University, Wuhan 430072, China; omarsalem@whu.edu.cn or; 2 Department of Information System, Faculty of Computers and Informatics, Suez Canal University, Ismailia 41522, Egypt; 3 Department of Computer Science and Information Technology, La Trobe University, Melbourne 3086, Australia; phoebe.chen@latrobe.edu.au

**Keywords:** feature selection, fuzzy sets, mutual information, rough set

## Abstract

The main challenge of classification systems is the processing of undesirable data. Filter-based feature selection is an effective solution to improve the performance of classification systems by selecting the significant features and discarding the undesirable ones. The success of this solution depends on the extracted information from data characteristics. For this reason, many research theories have been introduced to extract different feature relations. Unfortunately, traditional feature selection methods estimate the feature significance based on either individually or dependency discriminative ability. This paper introduces a new ensemble feature selection, called fuzzy feature selection based on relevancy, redundancy, and dependency (FFS-RRD). The proposed method considers both individually and dependency discriminative ability to extract all possible feature relations. To evaluate the proposed method, experimental comparisons are conducted with eight state-of-the-art and conventional feature selection methods. Based on 13 benchmark datasets, the experimental results over four well-known classifiers show the outperformance of our proposed method in terms of classification performance and stability.

## 1. Introduction

Nowadays, classification systems have a lot of contributions in different domains such as bioinformatics, medical analysis, text categorization, pattern recognition, and intrusion detection [1]. The main challenge of these systems is to deal with high dimensionality data, which may include redundant or irrelevant features [2]. These features have a negative effect on classification systems which can lead to (1) reducing the classification accuracy, (2) reducing the classification speed, (3) increasing the classification complexity. To overcome these limitations, features selection introduces an effective solution to reduce the dimensionality of data by selecting the significant features and discarding the undesirable ones [3].

Feature selection methods are divided into three categories: filter [4], embedded [5], and wrapper [6]. These methods can be also classified into two groups according to the role of classifiers in the feature selection process: classification-independent (filter method), and classification dependent group (embedded, and wrapper method) [3]. The former depends only on the data characteristics without considering classifiers in the selection process, while the latter depends on classifiers to assess the significance of features in the selection process. Although the classification-dependent group can return the best feature selection subset, it requires more computational cost as a result of the classification process. Moreover, the selected features related only to the used classifier in the feature selection process. For this reason, classification-independent is more practical for high dimensionality data [7]. In this study, filter feature selection is our interest rather than embedded and wrapper due to its benefits such as simplicity, practicality, scalability, efficiency, and generality [8].

The success of filter methods depends on the amount of extracted information from data characteristics [9]. Motivated by this hypothesis, many theories have been introduced to find the best filter feature selection method such as information theory [10], and rough set theory [11]. Information theory measures can rank the features not only according to their relevancy to class but also with respect to the redundancy of features [12]. Moreover, These measures outperform other measures as correlation due to its ability to deal with linear and non-linear relations [3]. Rough set theory can select a subset of features according to their dependency to class [13]. The main advantages of rough set measures are simplicity, and no user-defined parameter is required. However, the traditional measures of these theories share common limitation, they can not deal directly with continuous features. To overcome this limitation, many research studies have been extended by integrating the previous theories with fuzzy set theory [14,15,16]. Feature selection based fuzzy sets is not only suitable for any kind of data but also extracts more information from classes compared with the traditional feature selection methods [14]. In addition to its ability to deal with noise data [17].

Traditional methods based on previous theories estimate the feature significance based on either individually or dependency discriminative ability. Consequently, there is no general feature selection method, which returns the best feature subset with all datasets [18]. The traditional solution is to understand the data characteristics before the feature selection process. This solution is not efficient because of the high computational cost of expert analysis. To overcome this limitation, a new research direction, called an ensemble feature selection, is introduced, which combines more than one feature selection to cover all situations [2].

In this study, we propose a new ensemble feature selection method (fuzzy feature selection based on relevancy, redundancy, and dependency (FFS-RRD)) to utilize the previous theories. Firstly, we proposed a new method, called fuzzy weighted relevancy-based FS (FWRFS) to estimate the individually discriminative ability. Then, we combined it with fuzzy lower approximation-based FS (L-FRFS) to estimate the dependency discriminative ability [16]. The former method extracts two relations: relevancy and redundancy, while the latter extracts the dependency relation. The aim is to investigate these relations and produce a unique and effective feature selection method to improve classification methods.

The paper is organized as follows: Section 2 presents the main criteria of feature selection: relevancy, redundancy, and dependency. Then, the related work is presented in Section 3. Section 4 introduces the proposed method: fuzzy feature selection based on relevancy, redundancy, and dependency (FFS-RRD). After that, the experiment setup is showed in Section 5. Section 6 analyzes the experimental results. Finally, the conclusion is reported in Section 7.

## 2. Relevancy, Redundancy, and Dependency Measures

Filter-based FS methods try to find the best feature subset based on data characteristics without depending on classification models [4]. For this reason, they depend on the characteristics of data to find the most significant features. Consequently, filter-based feature selection methods study different data relations such as the relation between features and class, and the relation among features. There are three well-known feature relations: relevancy, redundancy, and dependency.

Firstly, relevancy relation measures the amount of shared information between features and the class [15]. However, some features may have the same relevancy relation and do not add new information to discriminate the classes. These features are considered redundant and no need to be selected. Redundancy relation measures the amount of shared information among features [15]. Another important feature relation is dependency [16]. Dependency relation measures the membership degree of feature subset to class. In the following, we present the definitions of these relations based on the fuzzy set theory [15,16].

Given a dataset D=(U,F∪C), where $U=\{u_{1},u_{2},\ldots ,u_{m}\}$ is a finite set of *m* instances, $F=\{f_{1},f_{2},\ldots ,f_{n}\}$ is a finite set of *n* features, and $C=\{c_{1},c_{2},\ldots ,c_{l}\}$ is a finite set of *l* classes. Let $f:U\to V_{f}$, where $V_{f}$ is the feature value on *U*. Every feature f∈F can be represented by fuzzy equivalence relation $E_{f}$ on *U* and defined by the following fuzzy relation matrix $M(E_{f})$.

(1) $$M\left(E_{f}\right)=\left(\begin{array}{cccc} e_{11} & e_{12} & \cdots & e_{1m}\\ e_{21} & \cdots & \cdots & e_{2m}\\ \cdots & \cdots & \cdots & \cdots \\ e_{m1} & e_{m2} & \cdots & e_{mm} \end{array}\right)$$

where $e_{ij}=E\left(x_{i},x_{j}\right)$ is the fuzzy equivalence relation that defines the similarity degree between $x_{i}$ and $x_{j}$, where $x_{i},x_{j}\in U$.

Fuzzy equivalence class ${\left[x_{i}\right]}_{E_{f}}$ of $x_{i}$ is defined by the following fuzzy set on *U*:

(2) $${\left[x_{i}\right]}_{E_{f}}=\frac{e_{i1}}{x_{1}}+\frac{e_{i2}}{x_{2}}+\cdots +\frac{e_{im}}{x_{m}}$$

Fuzzy entropy of feature *f* based on $E_{f}$ is defined as

(3) $$H\left(f\right)=\frac{1}{m}\sum_{i=1}^{m}\log\frac{m}{\left|{\left[x_{i}\right]}_{E_{f}}\right|}$$

where, the cardinal value of ${\left[x_{i}\right]}_{E_{f}}$ is defined as $|{\left[x_{i}\right]}_{E_{f}}|=\sum_{j=1}^{m}e_{ij}$.

Indiscernibility relation $IND(\bar{F})$ defines a set of objects that have the same equivalence class, where $\bar{F}\subseteq F$. The fuzzy partition of *U* on $IND(\bar{F})$ is defined by $U/IND\left(\bar{F}\right)=\left\{X_{1},X_{2},\ldots ,X_{\bar{F}}\right\}$, where $X_{k}=\left\{x_{j}\in U|{\left[x_{i}\right]}_{E}={\left[x_{j}\right]}_{E}\right\}$, and $x_{i}\in X_{x},k=\left\{1,2,\ldots ,\bar{F}\right\}$.

The fuzzy lower approximation of a single fuzzy equivalence class *X* is defined as

(4) $$\begin{array}{c} \mu_{\underline{E_{\bar{F}}X}}\left(x_{i}\right)=\underset{x_{j}\in U}{\inf}I\left(\mu_{E_{\bar{F}}}\left(x_{i},x_{j}\right),\mu_{X}\left(x_{j}\right)\right) \end{array}$$

where $\mu_{E_{\bar{F}}}\left(x_{i},x_{j}\right)={⋂}_{f\in \bar{F}}\mu_{E_{f}}\left(x_{i},x_{j}\right)$, and $I=min(1,1-x_{i}+x_{j})$ is the fuzzy Łukasiewicz implicator.

The fuzzy positive region determines all the objects on *U* that discriminate the classes of U/IND(C) based on a set of features $\bar{F}$. The fuzzy positive region is defined as

(5) $$\begin{array}{c} \mu_{POS_{E_{\bar{F}}}\left(C\right)}\left(x_{i}\right)=\underset{X\in U/C}{\sup}\mu_{\underline{E_{\bar{F}}}X}\left(x_{i}\right) \end{array}$$

### 2.1. Relevancy

Let $E_{f}$ and $E_{C}$ are two fuzzy relations of feature *f* and class *C* on *U*, respectively. Then, the fuzzy mutual information between *f* and *C* is defined as

(6) $$\begin{array}{c} I\left(f;C\right)=\frac{1}{m}\sum_{i=1}^{m}\log\frac{m|{\left[x_{i}\right]}_{E_{f}}\cap {\left[x_{i}\right]}_{E_{C}}|}{|{\left[x_{i}\right]}_{E_{f}}|.\left|{\left[x_{i}\right]}_{E_{C}}\right|} \end{array}$$

### 2.2. Redundancy

Let $E_{f_{1}}$ and $E_{f_{2}}$ be two fuzzy relations of features $f_{1}$ and $f_{2}$ on *U*, respectively. Then, the fuzzy mutual information between $f_{1}$ and $f_{2}$ is defined as

(7) $$\begin{array}{c} I\left(f_{1};f_{2}\right)=\frac{1}{m}\sum_{i=1}^{m}\log\frac{m|{\left[x_{i}\right]}_{E_{f_{1}}}\cap {\left[x_{i}\right]}_{E_{f_{2}}}|}{|{\left[x_{i}\right]}_{E_{f_{1}}}|.\left|{\left[x_{i}\right]}_{E_{f_{2}}}\right|} \end{array}$$

### 2.3. Dependency

Let $\bar{F}$ is a set of features, the dependency degree of $\bar{F}$ is defined as

(8) $$\begin{array}{c} {⋎}_{\bar{F}}\left(C\right)=\frac{\sum_{x_{i}\in U}\mu_{POS_{E_{\bar{F}}}\left(C\right)}\left(x_{i}\right)}{|{\left[x_{i}\right]}_{E_{f}}|} \end{array}$$

### 2.4. Example

To illustrate the computations of previous relations, a small example is presented in Table 1. Firstly, we estimate the relation matrix of each feature based on the following similarity equation [15]:

(9) $$E_{f}\left(x_{i},x_{j}\right)=\exp\left(-∥x_{i}-x_{j}∥\right)$$

As *C* contains discrete values, we estimate the relation matrix according to the crisp way [19].

The relation matrix of $f_{1}$ is:

$$\begin{array}{c} M\left(E_{f1}\right)=\left(\begin{array}{ccccc} 1.00 & 0.55 & 0.82 & 0.67 & 1.00\\ 0.55 & 1.00 & 0.67 & 0.82 & 0.55\\ 0.82 & 0.67 & 1.00 & 0.82 & 0.82\\ 0.67 & 0.82 & 0.82 & 1.00 & 0.67\\ 1.00 & 0.55 & 0.82 & 0.67 & 1.00 \end{array}\right) \end{array}$$

The relation matrix of $f_{2}$ is:

$$\begin{array}{c} M\left(E_{f2}\right)=\left(\begin{array}{ccccc} 1.00 & 0.67 & 0.82 & 0.74 & 1.00\\ 0.67 & 1.00 & 0.82 & 0.90 & 0.67\\ 0.82 & 0.82 & 1.00 & 0.90 & 0.82\\ 0.74 & 0.90 & 0.90 & 1.00 & 0.74\\ 1.00 & 0.67 & 0.82 & 0.74 & 1.00 \end{array}\right) \end{array}$$

The relation matrix of *C* is:

$$\begin{array}{c} M\left(E_{C}\right)=\left(\begin{array}{ccccc} 1.0 & 0.0 & 1.0 & 0.0 & 1.0\\ 0.0 & 1.0 & 0.0 & 1.0 & 0.0\\ 1.0 & 0.0 & 1.0 & 0.0 & 1.0\\ 0.0 & 1.0 & 0.0 & 1.0 & 0.0\\ 1.0 & 0.0 & 1.0 & 0.0 & 1.0 \end{array}\right) \end{array}$$

The fuzzy entropy of $f_{1}$ is:

$$\begin{array}{c} H\left(f_{1}\right)=\frac{1}{5}\left(\log\frac{5}{4.04}+\log\frac{5}{3.59}+\log\frac{5}{4.13}+\log\frac{5}{3.98}+\log\frac{5}{4.04}\right)=0.34 \end{array}$$

The fuzzy entropy of *C* is:

$$\begin{array}{c} H\left(C\right)=\frac{1}{5}\left(\log\frac{5}{3}+\log\frac{5}{2}+\log\frac{5}{3}+\log\frac{5}{2}+\log\frac{5}{3}\right)=0.97 \end{array}$$

The relevancy between $f_{1}$ and *C* is:

$$\begin{array}{c} I\left(f_{1},C\right)=\frac{1}{5}\left(\log\frac{5*2.82}{4.04*3}+\log\frac{5*1.82}{3.59*2}+\log\frac{5*2.64}{4.13*3}+\log\frac{5*1.82}{3.98*2}+\log\frac{5*2.82}{4.04*3}\right)=0.21 \end{array}$$

The redundancy between $f_{1}$ and $f_{1}$ is:

$$\begin{array}{c} I\left(f_{1},f_{2}\right)=\frac{1}{5}\left(\log\frac{5*4.04}{4.04*4.23}+\log\frac{5*3.59}{3.59*4.06}+\log\frac{5*4.13}{4.13*4.36}+\log\frac{5*3.98}{3.98*4.29}+\log\frac{5*4.04}{4.04*4.23}\right)=0.24 \end{array}$$

For the first object $x_{1}$ of $f_{1}$, the fuzzy lower approximation of a single fuzzy equivalence class X=1 is:

$$\begin{array}{c} \mu_{\underline{E_{f_{1}}X=1}}\left(x_{1}\right)=\underset{x_{j}\in U}{\inf}I\left(\mu_{E_{\bar{F}}}\left(x_{1},x_{j}\right),\mu_{X}=1\left(x_{j}\right)\right)=\inf\left\{I\left(1.00,1.0\right),I\left(0.55,0.0\right),I\left(0.82,1.0\right),I\left(0.67,0.0\right),I\left(1.00,1.0\right)\right\}=0 \end{array}$$

For the first object $x_{1}$ of $f_{1}$, The fuzzy lower approximation of a single fuzzy equivalence class X=0 is:

$$\begin{array}{c} \mu_{\underline{E_{f_{1}}X=0}}\left(x_{1}\right)=\underset{x_{j}\in U}{\inf}I\left(\mu_{E_{\bar{F}}}\left(x_{1},x_{j}\right),\mu_{X}=0\left(x_{j}\right)\right)=\inf\left\{I\left(1.00,0.0\right),I\left(0.55,1.0\right),I\left(0.82,0.0\right),I\left(0.67,1.0\right),I\left(1.00,0.0\right)\right\}=0.33 \end{array}$$

Similarly, for the remaining objects of $f_{1}$:

$$\begin{array}{c} \mu_{\underline{E_{f_{1}}X=1}}\left(x_{2}\right)=0.33,\mu_{\underline{E_{f_{1}}X=0}}\left(x_{2}\right)=0\\ \mu_{\underline{E_{f_{1}}X=1}}\left(x_{3}\right)=0.0,\mu_{\underline{E_{f_{1}}X=1}}\left(x_{3}\right)=0.18\\ \mu_{\underline{E_{f_{1}}X=1}}\left(x_{4}\right)=0.18,\mu_{\underline{E_{f_{1}}X=1}}\left(x_{4}\right)=0.0\\ \mu_{\underline{E_{f_{1}}X=1}}\left(x_{5}\right)=0.0,\mu_{\underline{E_{f_{1}}X=1}}\left(x_{5}\right)=0.33 \end{array}$$

The fuzzy positive region for the first object x=1 is:

$$\begin{array}{c} \mu_{POS_{E_{f_{1}}}\left(C\right)}\left(x_{1}\right)=\underset{X\in 1,0}{\sup}\mu_{\underline{E_{f_{1}}}X}\left(x_{1}\right)=\underset{X\in 1,0}{\sup}\left(0,1\right)=0.33 \end{array}$$

For the remaining objects, the fuzzy positive region are:

$$\begin{array}{c} \mu_{POS_{E_{f_{1}}}\left(C\right)}\left(x_{2}\right)=0.33\\ \mu_{POS_{E_{f_{1}}}\left(C\right)}\left(x_{3}\right)=0.18\\ \mu_{POS_{E_{f_{1}}}\left(C\right)}\left(x_{4}\right)=0.18\\ \mu_{POS_{E_{f_{1}}}\left(C\right)}\left(x_{5}\right)=0.33 \end{array}$$

The dependency degree of $f_{1}$ is:

$$\begin{array}{c} {⋎}_{f_{1}}\left(C\right)=\frac{\sum_{x_{i}\in U}\mu_{POS_{E_{f_{1}}}\left(C\right)}\left(x_{i}\right)}{\left|U\right|}=\frac{0.33+0.33+0.18+0.18+0.33}{5}=0.27 \end{array}$$

## 3. Related Works

Filter approach evaluates the feature significant based on the characteristics of data only with full independence of classification models [1]. Although the filter approach has many benefits over embedded and wrapper approaches, it may fail to find the best feature subset [20]. For this reason, a great research effort has been introduced to study the feature characteristics with the aim to find the significant features that improve classification models.

Among a variety of evaluation measures, mutual information (MI) has a popularity solution in feature selection based information theory due to its ability to define different relation of features such as relevancy, and redundancy. The main advantages of MI are [3]: (1) ability to deal with deal linear and non-linear relations among features; (2) ability to deal with both categorical and numerical features. In the past decades, MI has been used in many feature selection methods. Mutual information maximization (MIM) [21] defines the significance of features based on the relevancy relation. It suffers from the redundant features. After that, mutual information based feature selection (MIFS) [22] has been introduced and improved in MIFS-U [23] to define the significance of features based on both relevancy and redundancy relation. However, both methods require a predefined parameter to balance between the relevancy and redundancy relations. In [24], minimum redundancy maximum relevance (mRMR) proposes automatic value to estimate the predefined parameter of MIFS, and MIFS-U. In the literature, several feature selection methods have been proposed to find the best estimation of the relevancy and redundancy relations such as joint mutual information (JMI) [25], conditional mutual information maximization (CMIM) [26], joint mutual information maximization (JMIM) [27], and max-relevance and max-independence (MRI) [28]. However, previous studies of feature selection based mutual information do not consider the balance of selected/candidate feature relevancy relation. To avoid this limitation, Zhang et al. [29] has introduced a new method to keep the balance between the feature relevancy relations, called feature selection based on weighted relevancy (WRFS).

Another important solution in the filter approach is rough set which used to measure the dependency relation of features. Feature selection based rough set tries to find the minimal feature subset that maximizes the informative structure of all features (termed a reduct) [30]. The main advantages of the rough set are (1) analyzing only the hidden facts in data, (2) extracting the hidden knowledge of data without additional user-defined information, and (3) returning a minimal knowledge structure of data [19]. Many studies on feature selection based on rough set have been done. Rough Set Attribute Reduction (RSAR) defines the significance of a subset of features based on the dependency relation [31]. However, there is no guarantee to return the minimum feature subset. Han et al. [32] proposes an alternative dependency relation to reduce the computational cost of the feature selection process. Zhong et al. [33] defines the significance of the feature subset based on the discernibility matrix. However, it is impractical for high dimensionality data. In Entropy Based Reduction (EBR) [34], the significance of the feature subset is defined based on entropy which returns the maximum amount of information. In the literature of rough sets, further feature selection methods have been introduced such as Variable precision rough sets (VPRS) [35], and parameterized average support heuristic (PASH) [36].

However, both MI and rough set share common limitations when dealing with features of continuous values [19,37]. There are two traditional solutions have been proposed to overcome this limitation: parzen window [38], and discretization process [39]. The former has some limitations: firstly it requires a predefined parameter to compute the window function [40]. Secondly, it does not work efficiently with high dimensional data of spare samples [15]. The latter may lead to loss of feature information [41]. To overcome these limitations, FS based information theory and FS based rough set have been extended by fuzzy set theory to deal with continuous features directly [14,19]. However, most of FS methods based information theory focus on relevancy and redundancy relation, while FS methods based on rough set focus on dependency relation. The former depends on individually discriminative ability, while the latter depends on dependency discriminative ability. As a result, the traditional methods do not take the benefits of all types of discriminative ability.

## 4. Fuzzy Feature Selection Based on Relevancy, Redundancy, and Dependency (FFS-RRD)

**Figure 1:**
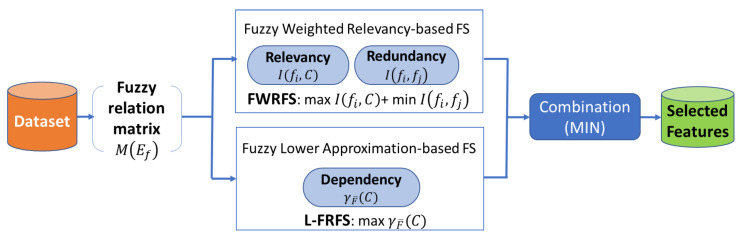
The process of our proposed method fuzzy feature selection based relevancy, redundancy, and dependency (FFS-RRD): firstly, the fuzzy relation matrix is generated for each feature in the dataset. Then, fuzzy mutual information maximizes the relevancy and minimizes the redundancy, while fuzzy rough set maximizes the dependency. Finally, the results are combined to find the selected features.

In this section, we present our proposed method, called FFS-RRD, as a filter feature selection method. The effectiveness of filter methods depends on the amount of extracted information from the data characteristics. To promote our proposed method, we used both individually and dependency discriminative ability based on three criteria: relevancy, redundancy, and dependency. FFS-RRD aims to maximize both relevancy and dependency relations and minimize the redundancy ones. To design our proposed method, firstly, we modified WRFS to overcome their limitations: (1) it can not deal with continuous features without the discretization process which may lead to loss of feature information. (2) WRFS does not consider dependency relation in the feature selection process. To overcome these limitations, we estimated WRFS based on the fuzzy concept instead of the probability concept. The extended method, called FWRFS, can deal with any numerical data without the discretization process. Then, we combined FWRFS with fuzzy-rough lower approximations (L-FRFS) [16] to extract the dependency relation. Consequently, we proposed a unique FS method, called FFSRRD, which maximizes both relevancy and dependency, and minimizes the redundancy relation. The three relations can extract more information from the dataset to promote the discriminate ability of feature selection. Figure 1 shows the process of the proposed method FFS-RRD. Both FWRFS and L-FRFS are applied on the same dataset. FWRFS selects the most relevant features and removes the redundancy ones, while L-FRFS selects the most dependency feature subset. The results of each method are combined to return the final feature selection subset. In our study, we used one of the popular combination methods called MIN [2]. MIN method assigns the minimum position of each feature among different results of feature selection methods to be ranked position in the final result.

The algorithm of the proposed method is presented in Algorithm 1. FFS-RRD depends on a combination of two methods: For the first method, FWRFS is used to return the ranked feature set that maximizes the relevancy and minimizes the redundancy. In the first step (Lines 1–3), The main parameters are initialized: ranked feature set ($R_{1}$), candidate feature (candidate), and the current selected feature set (selected). Then, the feature of maximum relevancy with class is selected to be the first ranked feature in $R_{1}$, and removed from the feature set *F* (Lines 4–8). After that, the feature of maximum relevancy with class and minimum redundancy with selected features is added to $R_{1}$, and removed from *F*. This process is repeated until all features of *F* are ranked in $R_{1}$ (Lines 9–14). For the second method, L-FRFS is used to return the subset of features that maximizes the dependency relation. In the first step (Lines 15–17), the main parameters are initialized: selected feature subset ($R_{2}$), temporary feature (*T*), maximum dependency degree (${⋎}_{select}$), and the last maximum dependency degree (${⋎}_{last}$). Then, the feature of maximum dependency is added to $R_{2}$. This process is repeated until the maximum possible dependency degree of features be produced (Lines 18–25). Finally, the result of both methods is combined by $MIN(R_{1},R_{2})$ to select the final feature subset (Line 26–27).
 **Algorithm 1:** FFS-RRD: fuzzy feature selection based relevancy, redundancy, and dependency. 
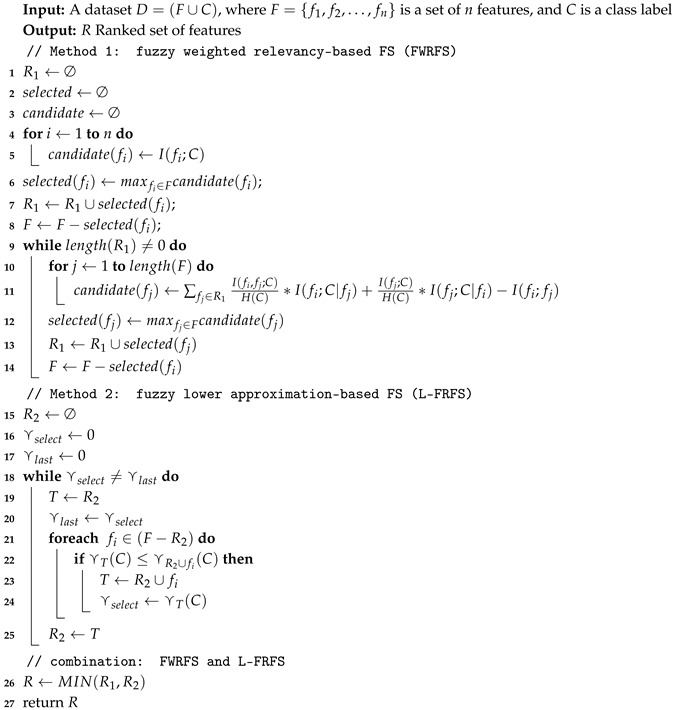


## 5. Experiment Setup

**Figure 2:**
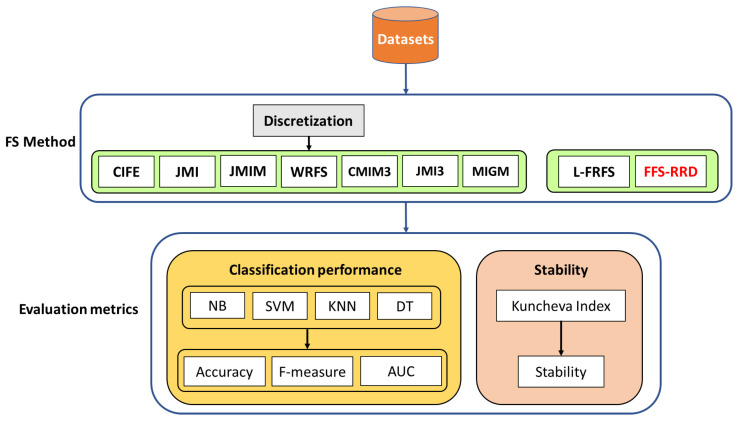
An experimental framework of the proposed method fuzzy feature selection based relevancy, redundancy, and dependency (FFS-RRD): firstly, a discretization process is applied before probability-based methods. Then, the compared methods are evaluated in terms of classification performance, and stability.

The main goal of the feature selection process is to improve the classification performance with the minimum feature selection subset. To validate our proposed method, we used four classifiers to compare the proposed method with eight feature selection methods based on benchmark datasets. Figure 2 shows the framework of our experiment. In the following, we present more details about the experiment setup.

### 5.1. Dataset

Our experiment was conducted based on 13 benchmark datasets from machine learning repository (UCI) [42]. The datasets support different classification problems of binary and multi-class data. Table 2 presents a brief description of the experimental datasets.

### 5.2. Compared Feature Selection Methods

Table 3 shows the compared FS methods and their discriminative ability. The compared methods can be divided into two groups: probability-based, and fuzzy-based. Firstly, probability-based group uses the probability concept to estimate information measures. The probability-based group consists of CIFE [43], JMI [25], JMIM [27], WRFS [29], CMIM3 [44], JMI3 [44], and MIGM [45]. This group depends on the discretization process before implementation of feature selection methods. In our experiment, the discretization process transforms the continuous features into discrete features with ten equal intervals [46].

Unlike a probability-based group which requires discretization preprocess, fuzzy-based group uses the fuzzy concept to estimate information measures. The fuzzy-based group includes L-FRFS [16], and the proposed method FFS-RRD. This group depends on similarity relation which transforms each feature into a fuzzy equivalence relation. In our experiment, we used the following similarity relation [15].

(10) $$E_{f}\left(x_{i},x_{j}\right)=\exp\left(-∥x_{i}-x_{j}∥\right)$$

### 5.3. Evaluation Metrics

The main factors characterize the quality of feature selection methods are its classification performance and stability [47]. The evaluation of our experiment is divided into two parts: classification performance and stability evaluation. Classification performance requires classification models to evaluate the effect of feature selection methods on improving the classification performance, while stability measures the robustness of feature selection methods.

#### 5.3.1. Classification Performance

To evaluate the classification performance, we used three metrics: classification accuracy, F-measure (β=1), AUC. The experiment depends on four classifiers: Naive bayes (NB), support vector machine (SVM), K-nearest neighbors (KNN, K = 3), and decision tree (DT). To find reliable results, we used 10-fold cross-validation where the dataset is divided into ten equal parts, nine for the training phase and one for the test phase [48]. This process is repeated ten times. Then, we calculate the average results to compute the score of accuracy, F-measure, and AUC.

In this experiment, we used a threshold to cut the ranked features and return a subset of selected features. The threshold is the median position of the ranked features (or the nearest integer position if the number of ranked features is even). For L-FRFS, we used the same threshold if the size of the returned subset is more than the median of all features.

#### 5.3.2. Stability Evaluation

The confidence of feature selection method is not only about the improvement of classification performance but also related to the robustness of the method [49]. The robustness of feature selection method against any small change of data, as a noise, is called feature selection stability [50]. In the stability experiment, we injected the data by 10% of noise which is generated based on standard deviation and the gaussian distribution of each feature [51]. Then, we run the feature selection method to return the sequence of features. This process is repeated for ten times with a new returned sequence each time. After that, we measure the stability for each feature selection method based on Kuncheva stability measure which is defined as [52]:

(11) $$Kun_{stab}=\frac{2}{p(p-1)}\sum_{i=1}^{p-1}\sum_{j=i+1}^{p}Kun_{index}\left(R_{i},R_{j}\right)$$

where *p* is the number of feature selection sequences, and $Kun_{index}\left(R_{i},R_{j}\right)$ is the Kuncheva stability index between two feature selection sequences $R_{i}$, and $R_{j}$ which is defined as:

(12) $$Kun_{index}\left(R_{i},R_{j}\right)=\frac{wn-r^{2}}{r(n-r)},$$

where $w=|R_{i}\cap R_{j}|$, $r=\left|R_{i}\right|=\left|R_{j}\right|$, and *n* is the total number of features.

## 6. Results Analysis

### 6.1. Classification Performance

#### 6.1.1. Accuracy

Based on NB classifier, it is obvious that FFS-RRD achieved the maximum average accuracy with score 83.4%, as shown in Table 4. The proposed method was more accurate than compared methods by the range from 0.4% to 1.8%. The order of methods ranked after FFS-RRD was JMIM, followed by JMI, both CMIM3 and JMI3, MIGM, WRFS, L-FRFS, and CIFE.

According to SVM classifier, FFS-RRD achieved the maximum average accuracy of all datasets by 86.4%, while L-FRFS achieved the minimum average accuracy by 84.1%, as shown in Table 5. The proposed method outperformed other methods in the range from 0.5% to 2.3%. The second-best feature selection method was JMI, followed by CMIM3, both JMIM and JMI3, WRFS, MIGM, and CIFE.

In the case of KNN classifier, FFS-RRD also was the best feature selection method in the term of average accuracy by 85.4%, while L-FRFS was the worst method by 82.5%, as shown in Table 6. After that, MIGM achieved the second-best method, followed by both JMI and JMIM, JMI3, WRFS, CMIM3, CIFE. The proposed method achieved better accuracy in the range from 0.5% to 2.9%.

Similarly, FFS-RRD kept the best average accuracy of DT classifier by 84.5%, as shown in Table 7. The proposed method outperformed other methods in the range from 0.4% to 1.4%. In contrast, both CIFE and L-FRFS achieved the worst results by 83.1%. The second-best feature selection method was JMI, followed by JMIM, both WRFS and JMI3, MIGM, and CMIM3.

#### 6.1.2. F-Measure

**Figure 3:**
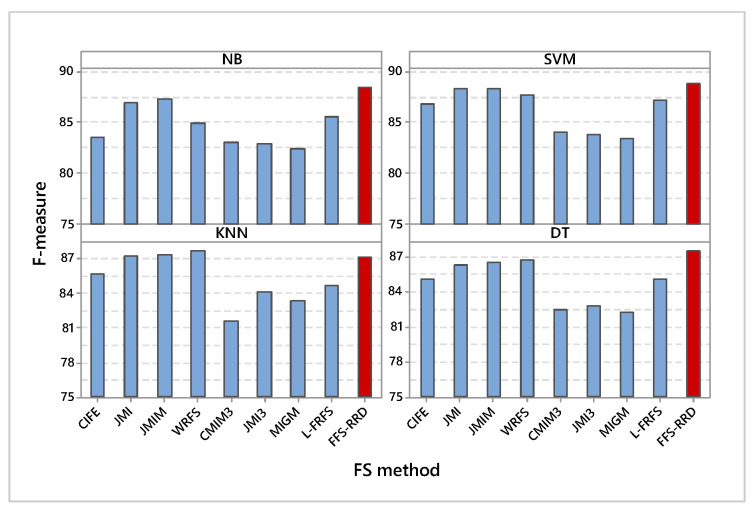
Average F-measure on the four used classifiers. Our proposed method (FFS-RRD) achieved the best result in all cases except KNN.

Figure 3 shows the F-measure of the compared methods based on the four used classifiers. In NB classifier, FSS-RRD achieved the maximum average F-measure by 88.5%, while MIGM achieved the minimum score by 82.4%. The proposed method outperformed other methods in the range from 1.5% to 6.1%. Similarly, FSS-RRD achieved the maximum average F-measure using SVM by 88.9%, while MIGM achieved the minimum score by 83.5%. The proposed method outperformed other methods in the range from 0.5% to 5.4%. According to KNN classifier, WRFS achieved the maximum average F-measure by 87.7%, while CMIM3 achieved the minimum score by 81.6%. The proposed method achieved the fourth-best position in this case. In DT classifier, FSS-RRD achieved the maximum average F-measure by 87.5%, while MIGM achieved the minimum score by 82.3%. The proposed method outperformed other methods in the range from 0.7% to 5.2%.

#### 6.1.3. AUC

**Figure 4:**
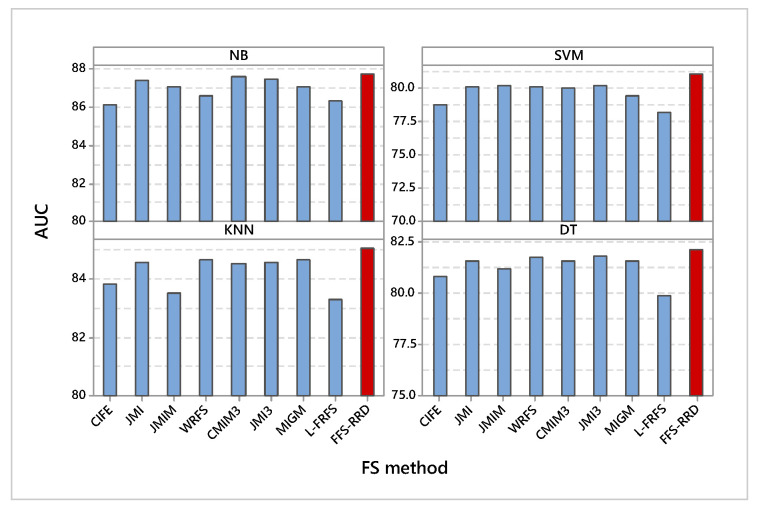
Average AUC on the four used classifiers. Our proposed method (FFS-RRD) achieved the best result in all cases.

It is obvious that the proposed method achieved the highest AUC compared with other methods using all classifiers (Figure 4). According to NB, FSS-RDD achieved the maximum AUC by 87.8%, while CIFE achieved the minimum AUC by 86.2%. The proposed method outperformed other methods in the range from 0.2% to 1.6%. In SVM classifier, FSS-RDD also achieved the maximum AUC by 81.1%, while L-FRFS achieved the minimum score by 78.2%. The proposed method outperformed other methods in the range from 0.9% to 2.9%. Similarly, FSS-RDD also achieved the maximum AUC using KNN by 85.1%, while L-FRFS achieved the minimum score by 83.3%. The proposed method outperformed other methods in the range from 0.4% to 1.8%. Using DT classifier, FSS-RDD kept the best method by 82.2%, L-FRFS kept the worst method by 79.9%. The proposed method outperformed other methods in the range from 0.4% to 2.3%.

**Figure 5:**
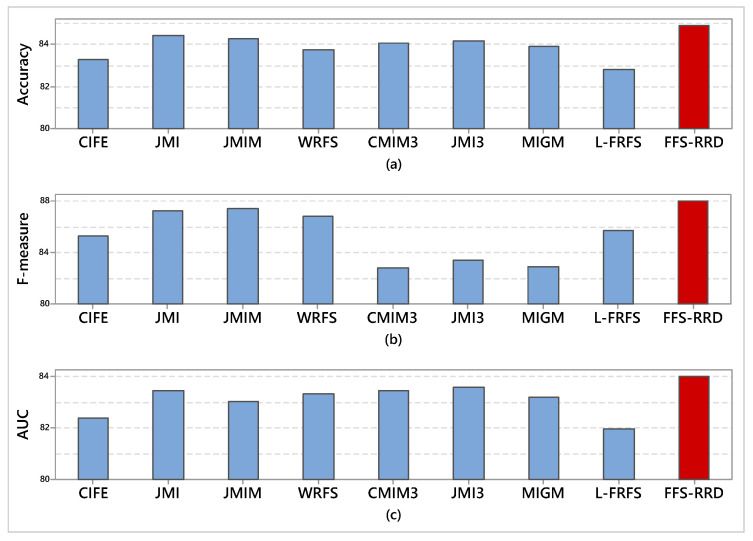
Average score of all classifiers in terms of accuracy, F-measure, and AUC. Our proposed method (FFS-RRD) achieved the best result.

Figure 5 shows the average score of the four classifiers in terms of accuracy, F-measure, and AUC. For accuracy term, FFS-RRD achieved the highest accuracy for all classifiers by 84.9%, followed by JMI, JMIM, JMI3, CMIM3, MIGM, WRFS, CIFE, and L-FRFS by 84.4%, 84.3%, 84.2%, 84.1%, 83.9%, 83.8%, 83.3%, and 82.8%, respectively. The proposed method outperformed other methods in the range from 0.5% to 2.1%. Similarly, FFS-RRD achieved the highest average of F-measure by 87.6%. Then, JMIM achieved the second-best method, followed by JMI, WRFS, L-FRFS, CIFE, JMI3, MIGM, CMIM3 with score 87.4%, 87.3%, 86.8%, 85.7%, 85.7%, 85.3%, 83.4%, 83.9%, 82.8%, respectively. The proposed method outperformed other methods in the range from 0.2% to 4.7%. According to AUC, FFS-RRD achieved the highest AUC with a score 84.0%. JMI3 achieved the second-best method, followed by JMI, CMIM3, WRFS, MIGM, JMIM, CIFE, and L-FRFS by 83.6%, 83.5%, 83.4%, 83.3%, 83.2%, 83.0%, 82.4%, and 81.9%, respectively. The proposed method outperformed other methods in the range from 0.4% to 2.1%.

### 6.2. Stability

**Figure 6:**
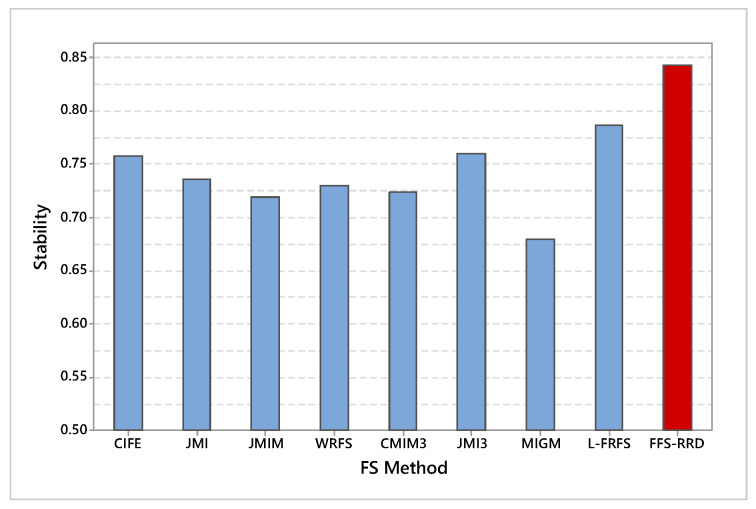
Average stability across the first half thresholds on all datasets. Our proposed method (FFS-RRD) achieved the best result.

**Figure 7:**
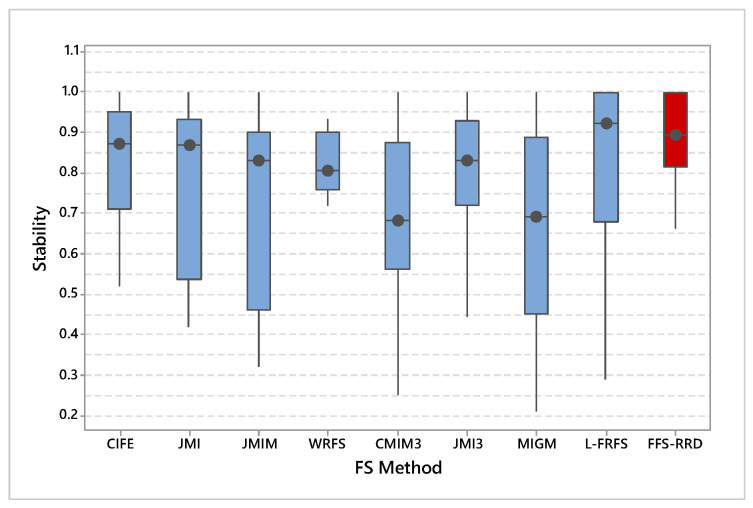
Average stability for all compared methods on the median threshold. Our proposed method (FFS-RRD) achieved the best result.

Figure 6 shows the average stability across the first half thresholds on all datasets. FFS-RRS achieved the maximum average of stability by 84.3%, while MIGM achieved the minimum score by 67.9%. After that, L-FRFS achieved the second-best method by 78.6%, followed by JMI3, CIFE, JMI, WRFS, CMIM3, and JMIM with an average score 76.0%. 75.8%, 73.5%, 73.0%, 72.3%, and 71.9%, respectively. The proposed method outperformed other methods in the range from 5.7% to 16.4%. Figure 7 shows a box-plot of average stability for all compared methods on the median threshold. In box-plot, the black circle represents the stability median, while the box represents both lower and upper quartiles. As shown in the box-plot, the stability result of the proposed method is better and more consistent than compared methods.

By considering the previous results, it is obvious that FFS-RRD achieved the best experimental results in the term of classification performance and feature stability. This is expected where the proposed method considers the individually and dependency discriminative ability of features. On the other hand, it is obvious that fuzzy-based methods are more stable than probability-based methods. The reason returns to using fuzzy sets to estimate the feature significance without information loss. Consequently, it helps fuzzy-based methods to be more stable against the noise.

## 7. Conclusions

In this paper, we have proposed an ensemble feature selection method, fuzzy feature selection based on relevancy, redundancy, and dependency criteria (FFS-RRD). Unlike the traditional methods, FFS-RRD depends on both individually and dependency discriminative ability. FFS-RRD aims to extract the significant relations from data characteristics to find the best feature subset that improves the performance of classification models. The proposed method consist of combination of two methods: FWRFS, and L-FRFS. FWRFS maximizes the relevancy and minimizes the redundancy relation, while L-FRFS maximizes the dependency relation.

Compared with eight state-of-the-art and conventional FS methods, experiments on 13 benchmark datasets indicate the outperformance of the proposed method in classification performance and stability. Classification performance includes three measures accuracy, F-measure, and AUC. The proposed method FFS-RRD achieved the highest average score of accuracy, and AUC on all datasets, while it achieved the highest average of F-measure on most of the classifiers except KNN classifier. On the other hand, the proposed method achieved the highest average of stability compared with other feature selection methods. In future work, we will extend the proposed method to explore their effect on multi-label classification models.

## Figures and Tables

**Table 1:** An example of a small dataset, contains two features ($f_{1},f_{2}$), and class *C*.

$f_{1}$	$f_{2}$	*C*
0.2 0.8 0.4 0.6 0.2	0.1 0.5 0.3 0.4 0.1	1 0 1 0 1

**Table 2:** Description of datasets used in the experiments.

Dataset	Brief	# Instances	# Features	# Classes
Breast Cancer Wisconsin (Prognostic)	BCW Prognostic	198	33	2
Breast Cancer Wisconsin (Diagnostic)	BCW Diagnostic	569	31	2
Climate Model Simulation Crashes	CMSC	540	18	2
Credit Approval	Credit Approval	690	15	2
Dermatology	Dermatology	336	34	6
Diabetic Retinopathy Debrecen	DRD	1151	19	2
Fertility	Fertility	100	9	2
Statlog (Heart)	Heart	270	13	2
Ionosphere	Ionosphere	351	34	2
Iris	Iris	150	4	3
Libras Movement	Libras Movement	360	90	15
QSAR biodegradation	QSAR	1055	41	2
Zoo	Zoo	101	16	7

**Table 3:** The extracted feature relations of compared feature selection methods.

Ref.	FS Group	FS Method	Discriminative Ability	
			Individually	Dependency
[43]	Probability-based	CIFE	✓	
[25]		JMI	✓	
[27]		JMIM	✓	
[29]		WRFS	✓	
[44]		CMIM3	✓	
[44]		JMI3	✓	
[45]		MIGM		✓
[16]	Fuzzy-based	L-FRFS		✓
Proposed		FFS-RRD	✓	✓

**Table 4:** Average classification accuracy on Naive bayes (NB) classifier: our proposed method achieved the best result.

Dataset	CIFE	JMI	JMIM	WRFS	CMIM3	JMI3	MIGM	L-FRFS	FFS-RRD
BCW Prognostic	73.9	69.5	73.8	65.4	68.6	68.8	67.7	71.3	69.8
BCW Diagnostic	92.0	93.4	93.4	93.6	93.2	92.4	92.9	85.9	93.6
CMSC	91.9	93.8	91.9	94.1	93.6	92.8	93.8	93.7	93.8
Credit Approval	85.6	83.5	83.7	86.9	82.4	83.7	84.8	76.8	85.4
Dermatology	96.1	93.9	95.3	93.1	98.0	94.6	95.3	96.2	96.1
DRD	57.6	60.3	57.6	57.5	57.7	60.6	60.3	57.6	57.5
Fertility	87.9	87.9	88.0	88.0	88.0	88.0	87.9	88.0	88.0
Heart	80.1	83.0	81.0	84.1	83.0	83.9	80.1	75.1	81.5
Ionosphere	77.0	82.6	86.2	80.8	84.8	84.8	77.4	89.8	89.0
Iris	94.6	94.6	94.6	94.6	93.5	93.5	93.5	95.0	95.0
Libras Movement	51.4	61.7	60.8	50.5	59.0	59.0	59.4	61.0	60.0
QSAR	78.2	78.7	77.1	78.5	78.7	78.1	77.6	77.7	80.0
Zoo	94.2	95.1	96.0	96.0	96.1	96.0	96.9	93.5	94.9
**Average**	81.6	82.9	83.0	81.8	82.8	82.8	82.1	81.7	83.4

**Table 5:** Average classification accuracy on support vector machine (SVM) classifier: our proposed method achieved the best result.

Dataset	CIFE	JMI	JMIM	WRFS	CMIM3	JMI3	MIGM	L-FRFS	FFS-RRD
BCW Prognostic	76.3	76.8	77.9	76.3	76.4	77.7	76.6	76.3	77.5
BCW Diagnostic	96.3	97.3	97.3	97.5	96.9	95.0	95.6	88.4	96.3
CMSC	91.5	92.0	91.5	93.2	91.9	91.6	91.9	92.1	91.9
Credit Approval	85.5	85.5	85.5	85.5	85.5	85.5	85.5	73.7	85.5
Dermatology	95.8	95.5	95.8	94.2	98.2	96.8	95.7	96.8	96.5
DRD	68.0	67.2	68.0	67.7	67.5	67.0	67.2	68.0	67.7
Fertility	88.0	88.0	88.0	88.0	88.0	88.0	88.0	88.0	88.0
Heart	79.7	84.3	84.3	84.2	81.3	83.7	79.7	76.3	82.5
Ionosphere	76.6	79.0	78.5	81.2	81.7	82.9	78.0	86.7	87.6
Iris	95.7	95.7	95.7	95.9	93.9	93.9	93.9	94.5	95.0
Libras Movement	72.2	76.1	74.7	67.6	75.5	74.9	74.5	76.5	75.3
QSAR	84.2	84.3	84.5	82.3	84.2	83.7	84.4	83.8	84.1
Zoo	92.8	95.3	88.7	93.3	93.9	89.5	94.1	92.0	95.3
**Average**	84.8	85.9	85.4	85.1	85.8	85.4	85.0	84.1	86.4

**Table 6:** Average classification accuracy on K-nearest neighbors (KNN) classifier: our proposed method achieved the best result.

Dataset	CIFE	JMI	JMIM	WRFS	CMIM3	JMI3	MIGM	L-FRFS	FFS-RRD
BCW Prognostic	75.2	75.0	77.1	76.6	73.5	74.1	76.9	74.0	77.0
BCW Diagnostic	93.3	93.5	97.1	95.4	95.1	94.6	95.2	87.5	94.8
CMSC	90.1	92.3	90.1	92.0	90.9	92.4	92.6	93.4	93.0
Credit Approval	84.7	83.9	85.1	85.5	84.4	85.3	85.1	76.4	84.6
Dermatology	95.0	95.9	95.6	93.7	97.7	96.9	96.1	96.1	96.3
DRD	64.3	63.7	64.3	64.1	62.3	63.7	63.6	64.7	64.1
Fertility	88.7	88.7	87.2	86.7	84.0	90.5	88.7	85.0	89.4
Heart	77.7	79.1	79.1	81.7	79.3	77.7	77.6	71.7	78.2
Ionosphere	81.8	80.9	80.3	83.0	82.3	83.3	83.5	82.7	84.2
Iris	93.9	93.9	93.9	93.9	91.8	91.8	91.8	92.7	92.7
Libras Movement	70.7	76.8	77.9	70.4	77.1	74.7	78.3	75.8	78.3
QSAR	84.0	84.7	83.3	81.5	83.8	84.4	83.6	82.5	83.0
Zoo	90.1	94.1	91.6	93.1	92.1	92.1	91.2	89.7	94.3
**Average**	83.8	84.8	84.8	84.4	84.2	84.7	84.9	82.5	85.4

**Table 7:** Average classification accuracy on decision tree (DT) classifier: our proposed method achieved the best result.

Dataset	CIFE	JMI	JMIM	WRFS	CMIM3	JMI3	MIGM	L-FRFS	FFS-RRD
BCW Prognostic	73.8	73.7	73.1	72.5	72.8	73.0	73.0	76.6	73.2
BCW Diagnostic	93.7	94.5	94.1	94.2	94.0	93.5	93.5	88.6	94.6
CMSC	89.6	91.2	89.6	90.3	91.4	91.2	91.3	91.3	91.6
Credit Approval	85.5	85.5	85.7	86.3	84.8	85.7	85.3	75.3	86.3
Dermatology	93.6	92.3	92.2	91.3	93.5	94.1	92.5	94.3	94.9
DRD	68.0	65.8	68.0	67.7	65.0	66.8	65.9	67.7	67.6
Fertility	87.1	87.1	87.1	87.6	86.6	86.6	87.1	87.5	87.5
Heart	73.9	80.3	81.3	80.6	76.3	77.6	74.0	74.3	75.5
Ionosphere	88.4	88.4	88.4	89.7	88.8	88.4	88.0	88.6	89.0
Iris	90.8	90.8	90.8	90.8	91.9	91.9	91.9	91.7	91.7
Libras Movement	60.4	66.0	65.6	61.3	64.7	64.3	67.5	66.0	66.4
QSAR	83.2	83.7	82.5	83.5	82.9	83.5	82.3	82.3	82.9
Zoo	91.9	94.0	92.3	92.9	93.4	92.1	94.1	96.0	97.1
**Average**	83.1	84.1	83.9	83.7	83.5	83.7	83.6	83.1	84.5
